# BCL2L10 is a predictive factor for resistance to Azacitidine in MDS and AML patients

**DOI:** 10.18632/oncotarget.481

**Published:** 2012-05-09

**Authors:** Thomas Cluzeau, Guillaume Robert, Nicolas Mounier, Jean Michel Karsenti, Maeva Dufies, Alexandre Puissant, Arnaud Jacquel, Aline Renneville, Claude Preudhomme, Jill-Patrice Cassuto, Sophie Raynaud, Frederic Luciano, Patrick Auberger

**Affiliations:** ^1^ INSERM U1065, Centre Mediterranéen de Médecine Moléculaire, Team «Cell Death, Differentiation, Inflammation and Cancer», Nice, France; ^2^ Université de Nice, France; ^3^ Equipe labellisée par la Ligue Nationale Contre le Cancer (2011-2013), Paris; ^4^ CHU de Nice, Service d'Hématologie Clinique, France; ^5^ CHU de Nice, Laboratoire d'Onco-hématologie, France; ^6^ CHRU de Lille, Centre de Biologie-Pathologie, Laboratoire d'Hématologie, France

**Keywords:** MDS, Azacitidine, resistance, BCL2L10, prognosis

## Abstract

Azacitidine is the leading compound to treat patients suffering myelodysplastic syndrome (MDS) or AML with less than 30% of blasts, but a majority of patients is primary refractory or rapidly relapses under treatment. These patients have a drastically reduced life expectancy as compared to sensitive patients. Therefore identifying predictive factors for AZA resistance is of great interest to propose alternative therapeutic strategies for non-responsive patients. We generated AZA-resistant myeloid cell line (SKM1-R) that exhibited increased expression of BCL2L10 an anti-apoptotic Bcl-2 family member. Importantly, BCL2L10 knockdown sensitized SKM1-R cells to AZA effect suggesting that increased BCL2L10 expression is linked to AZA resistance in SKM1-R. We next established in 77 MDS patients that resistance to AZA is significantly correlated with the percentage of MDS or AML cells expressing BCL2L10. In addition, we showed that the proportion of BCL2L10 positive bone marrow cells can predict overall survival in MDS or AML patients. We propose a convenient assay in which the percentage of BCL2L10 expressing cells as assessed by flow cytometry is predictive of whether or not a patient will become resistant to AZA. Therefore, systematic determination of BCL2L10 expression could be of great interest in newly diagnosed and AZA-treated MDS patients.

## INTRODUCTION

Azacitidine (AZA) is a hypomethylating agent approved for the treatment of patients suffering myelodysplastic syndromes[1] (MDS) and acute myeloid leukemia (AML) with low (20–30%) bone marrow blast counts in USA, Europe and other countries yielding 40–60% response in this disease[2, 3]. Current prognostic scoring systems consider karyotype abnormalities and certain clinical features to stratify patients with MDS into risk groups[4]. Thus, update of prognostic scoring is mainly based on new cytogenetic subgroups[5]. Cytogenetic findings can predict and are prognostic for overall survival (OS) in patients treated with hypomethylating agents[6, 7] but are relatively poor predictors of response[8]. Half of patients with MDS exhibit a normal karyotype, and patients with identical chromosomal abnormalities are often clinically heterogeneous[5, 9]. Somatic point mutations are common in MDS. Mutations in *TP53*, *EZH2*, *ETV6*, *RUNX1*, *DNMT3A* and *ASXL1* are predictors of poor OS in patients with MDS independently of other established risk factors[9-11]. Genetic alterations of the major splicing components including SF3B1 have been also reported in MDS[12-14]. However, prognostic impact depending on the treatment of all these mutations was not evaluated in this cohort of patients. To date, only mutations in TET2 have been identified as genetic predictors of response to AZA[15].

MDS or AML patients treated with AZA are either primary refractory (AZA-resistant) or AZA-sensitive but systematically relapse upon treatment with various time lapses[2]. Globally, only 17% of complete remission is observed with AZA treatment. Presence of partial remission and stable disease with hematologic improvement showed an increasing of OS in MDS or AML patients treated by AZA. Therefore, relapse or refractory patients are defined by presence of progression or stable disease without hematologic improvement according to IWG 2006 criteria. Outcome of MDS patient after AZA treatment failure is poor with a median overall survival of 5.6 months[16]. Importantly, no consensus genetic predictor of response to AZA or relapse after initial AZA sensitivity has been reported so far. Therefore, it seems of great importance to identify as early as possible those MDS patients treated by hypomethylating agents that will relapse inexorably in order to propose other clinical trials before worsening of clinical conditions.

We recently generated AZA-resistant SKM1 myeloid cells following long-term incubation with increasing concentrations of AZA. These cells exhibited impaired apoptosis in response to AZA[17]. In the present study, taking opportunity of the availability of this cell line model, we identify a new potential prognostic factor for the response to AZA in MDS. Indeed, we show for the first time that protein expression of BCL2L10, an anti-apoptotic member of the Bcl2 family is increased and correlated with AZA resistance in the AZA-resistant SKM1 cell line and that the percentage of BCL2L10 positive cells MDS primary sample patients can predict AZA resistance. We propose that systematic determination of the percentage of BCL2L10 positive cells by flow cytometry could be of great interest before treating MDS or AML patients with AZA. Moreover, evaluation of an increase in the proportion of BCL2L10 positive MDS cells could be also interesting in the course of AZA treatment.

## RESULTS

### Validation of a flow cytometry-based assay for BCL2L10 detection

**Figure 1:**
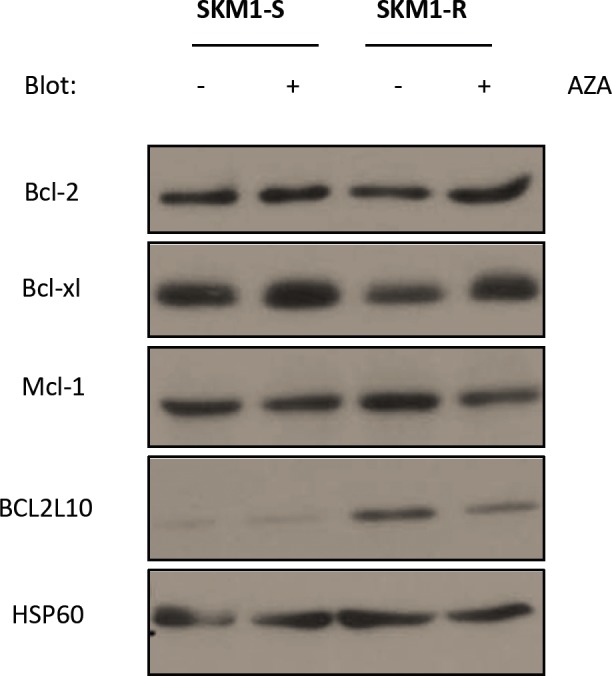
Expression of anti-apoptotic Bcl2 family members in SKM1-sensitive and resistant cell lines SKM1-S and SKM1-R were treated for 24h with 1μM AZA and Western Blots were carried out to assess Bcl-2, Mcl-1, Bcl-xL and BCL2L10 protein expression. HSP60 was used as loading control.

We recently generated AZA-resistant SKM1 cells (SKM1-R) defective for AZA-induced apoptosis[17]. Compared to their AZA-sensitive counterpart SKM1-R cells exhibited increased protein expression of BCL2L10 (Bcl-B), an anti-apoptotic member of the Bcl-2 family but equivalent levels of Bcl-2, Bcl-xL and Mcl-1 proteins (Figure 1). Increased BCL2L10 protein expression was also found in the SKM1-R bulk before limited dilution and also in another SKM1-R subclone (not shown), indicating that overexpression of BCL2L10 is linked to AZA resistance and is not due to a clonal effect. To analyze BCL2L10 protein expression, we developed a cytometry-based assay in HEK293 cells. HEK293 cells were first transfected with a tagged-Myc construct as a negative control or a tagged-Myc-BCL2L10 construct and transfection efficiency was assessed using an anti-BCL2L10 antibody. Endogenous BCL2L10 protein was detected in HEK293 cells transfected with the tagged-Myc construct (Figure S1A, curve 2) whereas a stronger staining was visualized in HEK293 cells overexpressing BCL2L10 as expected (Figure S1A, curve 3). BCL2L10 protein overexpression was confirmed by western blot using an anti-BCL2L10 mAb (Figure S1B). To validate the flow cytometry assay, we next used a specific BCL2L10 siRNA to knockdown BCL2L10 expression in HEK293 cells (Figure S1C). In this condition and conversely to the situation in which a control Luc siRNA was used (Figure S1C, curve 1), neither BCL2L10 protein expression nor BCL2L10 staining were detected by flow cytometry (Figure S1C, curve 2) and western blot (Figure S1D) validating our flow cytometry-based assay for BCL2L10 detection.

### Overexpression of BCL2L10 participates to AZA resistance in SKM1 cells

**Figure 2:**
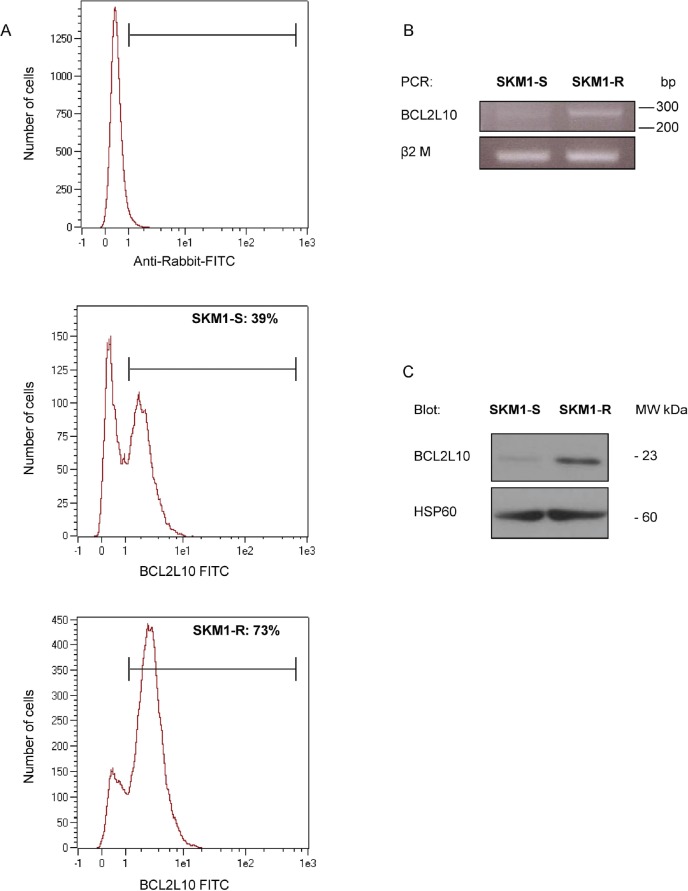
BCL2L10 protein expression in SKM1-S and SKM1-R cell lines (A) BCL2L10 protein level was quantified by flow cytometry in SKM1-S and SKM1-R cells as described in Supplementary Figure 1. (B) RT-PCR analysis of BCL2L10 mRNA expression in SKM1-S and SKM1-R cells. (C) BCL2L10 protein level was vizualized by Western Blot in SKM1-S and SKM1-R cells.

Using the same assay, we established that significantly more SKM1-R cells expressed BCL2L10 protein compared to SKM1-S (73% versus 39%) and that globally BCL2L10 expression was increased in SKM1-R cells (Figure 2A). An increased expression of both BCL2L10 mRNA and protein levels was also detected in SKM1-R cells as judged by RT-PCR (Figure 2B) and western blot (Figure 2C).

**Figure 3:**
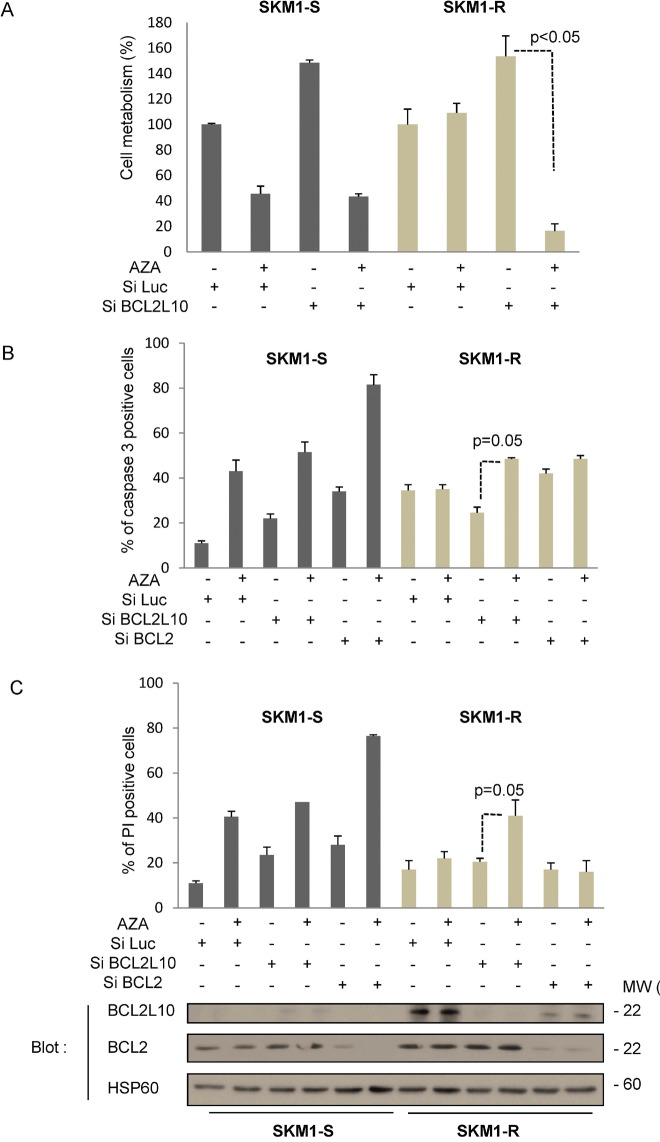
BCL2L10 knockdown sensitizes SKM1-R to the effect of AZA SKM1-S and SKM1-R cells were transfected with either control Luc, BCL2L10 or a Bcl-2 siRNAs. 72h after transfection, cells were stimulated with 1μM AZA. (A) Cell metabolism was assessed 24h later using the XTT assay as described in the material and methods section. Results represent the mean ± SEM of three independent experiments made in quadruplicates. Active caspase 3 (B) and propidium iodide staining (C) were visualized by flow cytometry 24h after the addition of 1μM AZA. (D) Western Blots were also carried out at the same time to assess BCL2L10 and Bcl-2 expression.

To determine whether BCL2L10 overexpression is the cause rather than a consequence of resistance to AZA, SKM1-S and SKM1-R cells were transfected with either a control siRNA or a siRNA directed against BCL2L10 and next treated for 24 h with or without AZA, before determination of cell viability and apoptosis. AZA triggered a loss of cell metabolism in SKM1-S but not in SKM1-R cells (Figure 3A), as expected. Knockdown of BCL2L10 restored sensitivity to AZA in SKM1-R suggesting an important role for BCL2L10 in resistance to AZA. For an unknown reason cell metabolism was higher in SKM1-S and SKM1-R cells treated with the BCL2L10 siRNA (Figure 3A). In addition, apoptosis was the main mechanism by which BCL2L10 knockdown mediated sensitization to AZA since both an increase in active caspase 3 and PI positive cells were detected in SKM1-R treated with a BCL2L10 siRNA (Figure 3B and C). This effect was specific for BCL2L10 because a siRNA directed against Bcl-2 failed to do so in identical conditions (Figure 3B and C). Of note, it should be pointed out that SKM1-R had higher active caspase 3 levels than their SKM1-S counterpart (Figure 3B and C). Finally we checked by western blotting that both siRNAs were able to abolish expression of their respective targets (Figure 3D). Collectively, our findings established that BCL2L10 protein overexpression is responsible for AZA resistance in SKM1-R cells.

### BCL2L10 expression is predictive of resistance to AZA in MDS patients

**Figure 4:**
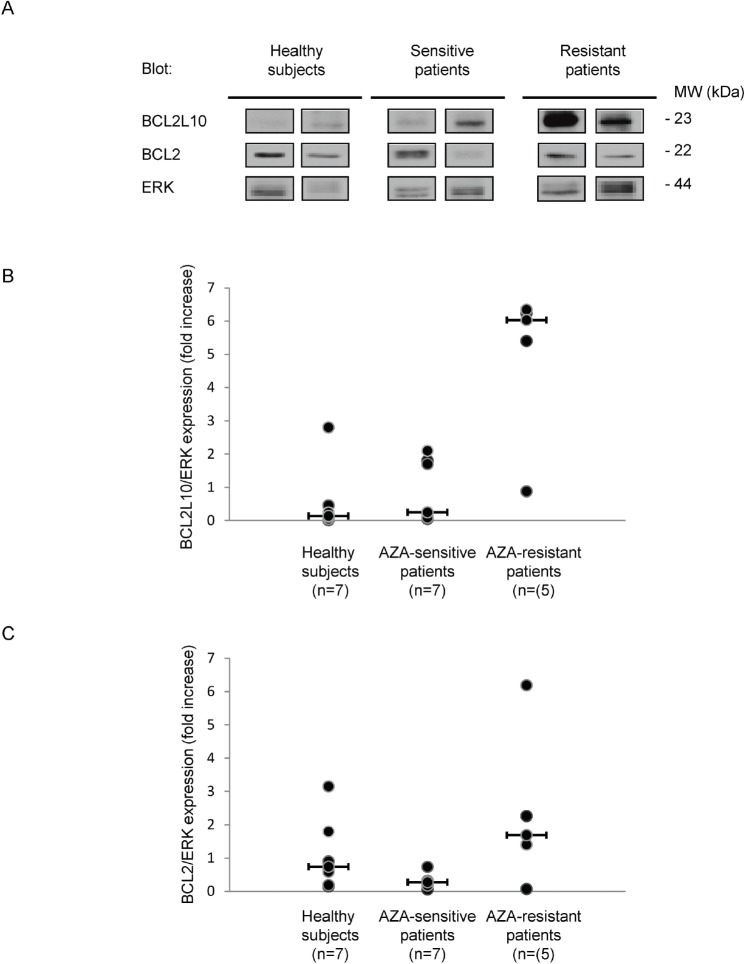
BCL2L10 protein expression is increased in AZA-resistant patients BCL2L10, Bcl-2 and ERK expression was analyzed by Western Blot on fresh bone marrow samples prepared from 7 healthy subjects, 7 AZA-sensitive and 5 AZA-resistant patients. (A) A Western Blot is shown for two representative patients of each subgroup. (B and C) BCL2L10, Bcl-2 and ERK quantification was performed using the Image J software and the ratio of BCL2L10 or Bcl-2 versus ERK expression were quantified.

We also analyzed BCL2L10 expression by western blot on patient samples, when sufficient material was available. Results presented on Figure 4 revealed that the level of BCL2L10 tends to be higher in two representative AZA-resistant patients as compared to two representative AZA-sensitive or healthy patients (Figure 4A). Using ERK protein as an internal control for each patient sample, we showed that BCL2L10 versus ERK protein expression was drastically higher in 5 AZA-resistant versus 7 AZA-sensitive patients, whereas the level of BCL2L10 versus ERK protein expression was very low in 7 healthy subjects (Figure 4B). Conversely, Bcl-2 protein expression was not significantly different in the three groups of patients (Figure 4C). Collectively, these results suggest that BCL2L10 expression is predictive of AZA-resistance in MDS patients.

### BCL2L10 protein expression as a biomarker of AZA resistance in MDS patients

**Figure 5:**
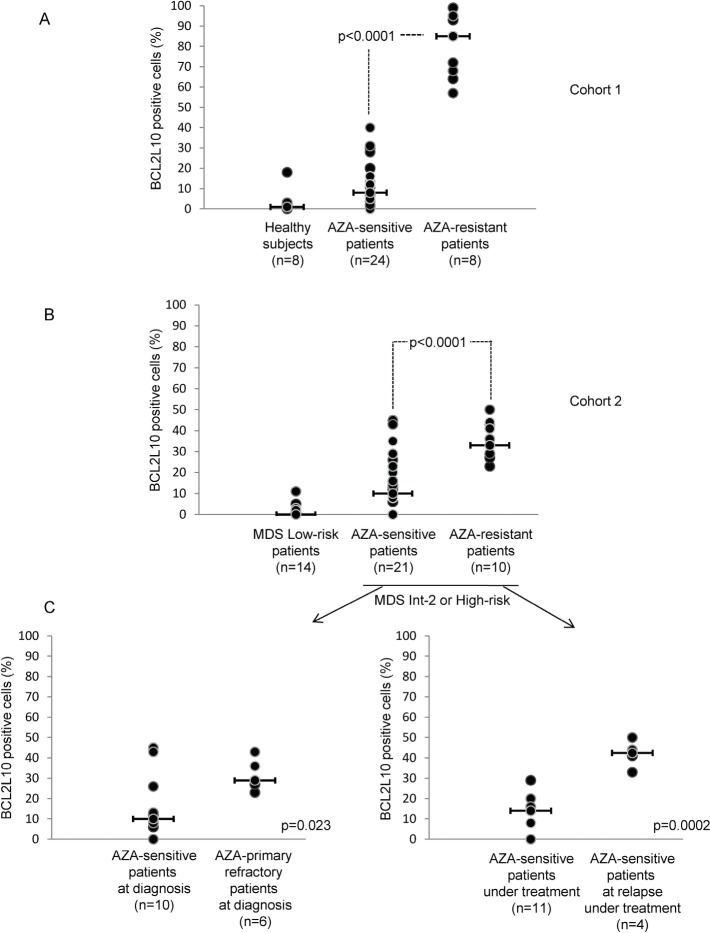
AZA-R MDS or AML patients treated with AZA have increased percentage of BCL2L10 expressing cells in their bone marrow (A) The percentage of BCL2L10 positive cells was quantified by flow cytometry in 32 MDS or AML patients treated with AZA and in 8 healthy subjects (cohort 1). (B) The percentage of BCL2L10 positive cells was quantified by flow cytometry in DMSO-frozen samples from 14 low-risk MDS patients, 31 high-risk MDS or AML patients treated with AZA (cohort 2). (C) Subgroups analysis of patients from cohort 2 was also performed (Figure 5B). The percentage of BCL2L10 positive cells was quantified in DMSO-frozen samples from 16 MDS high-risk patients or AML patients at diagnosis (left panel) and in DMSO-frozen samples from 15 MDS high-risk or AML patients under AZA treatment (right panel).

We next determined the percentage of BCL2L10 positive cells in freshly isolated bone marrow (BM) samples from 8 healthy subjects, 24 AZA-sensitive patients and 8 AZA-resistant patients (Cohort 1, fresh samples) using our cytometry-based assay. The clinical features of all patients are given in supplemental Table S1 and S2. As shown on Figure 5A the mean values for freshly isolated BM samples from healthy subjects and AZA-sensitive patients were respectively 0% (0-18) and 8% (0-40) BCL2L10 positive cells, whereas the mean value for BM cells from AZA-resistant patients was 85% (57-99) BCL2L10 positive cells (p<0.0001) (Figure 5A). In a second cohort (cohort 2 / frozen samples), retrospective comparison of BM samples from low risk MDS patients (n=14), AZA-sensitive (n=21) or AZA-resistant patients (n=10) showed that the counts of BCL2L10 positive cells of low risk MDS patients was 0% (0-11), whereas AZA-resistant patients had significantly higher numbers of BCL2L10 positive cells as compared to AZA-sensitive patients (33% versus 10%, p<0.0001) (Figure 5B).

In addition, we performed subgroups analysis from the 21 AZA-sensitive patients and the 10 AZA-resistant patients from cohort 2 (Figure 5B). AZA-primary resistant patients (n=6) had significantly higher counts of BCL2L10 positive cells (29%) than AZA-sensitive patients at diagnosis (10%) (n=6) (Figure 5C, left panel) (p=0.023). AZA-sensitive patients at relapse (n=4) exhibited high counts of BCL2L10 positive cells (43%), as compared to AZA-sensitive patients (n=11) under treatment (14%) (Figure 5C, right panel) (p=0.0002).

Finally, we analyzed in two patients (#4) and (#23), the parallel evolution of bone marrow blast counts and the percentage of BCL2L10 positive cells. After 9 cycles of AZA, patient #23 was sensitive to AZA, had 12% blasts (RAEB-II in the MDS classification) and 40% BCL2L10 positive cells (Figure S2A). This patient remains sensitive after 12 and 15 cycles of AZA. When this patient became resistant after 18 cycles of AZA, its blast counts and BCL2L10 positivity increased to 35% and 90%, respectively. These results suggest that BCL2L10 expression is a hallmark of resistance to AZA. In contrast, patient #4 who remained sensitive to AZA all along the treatment (over 29 cycles), failed to exhibit any increase in blast counts and in the number of BCL2L10 positive cells (Figure S2B).

### The percentage of BCL2L10 positive cells predicts OS in MDS and AML patients

**Figure 6:**
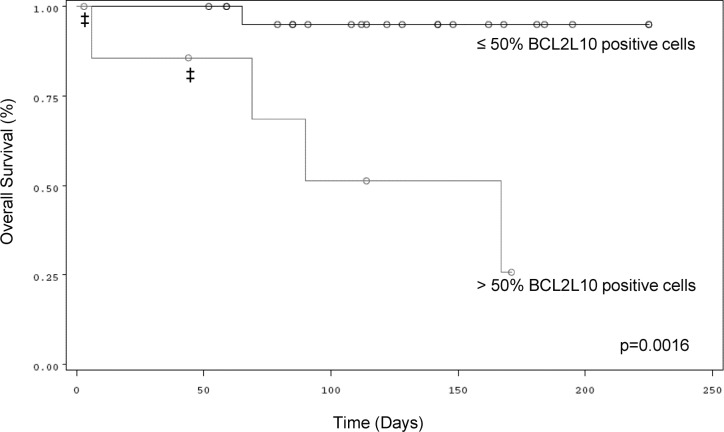
Correlation between the percentage of BCL2L10 expressing cells and OS in MDS or AML patients treated with AZA Kaplan-Meier OS curves of AZA-treated MDS or AML patients with more or less than 50% BCL2L10 expressing cells in their bone marrow. ‡ indicate allografted patients

We established that a cut-off of 50% BCL2L10 positive cells discriminates well between AZA-sensitive and resistant patients. Using this cut-off and the patients from cohort 1, we established that the test had excellent positive and negative predictive values. Globally, the test sensitivity and specificity was 80% and 85%. With a median follow-up of 4 months (range, 0.1 to 7.5 months) from the date of quantification of BCL2L10 positive cells, OS was significantly better in patients of cohort 1 exhibiting low percentage of BCL2L10 positive cells versus patients exhibiting high percentage of BCL2L10 positive (p=0.0016) (Figure 6). Estimated 3-month OS was 95% versus 51% in the subgroup of patients with low percentage of BCL2L10 positive cells compared to the subgroup of patients with high percentage of BCL2L10 positive cells, respectively. Importantly, all patients with a high percentage of BCL2L10 positive cells progressed rapidly. Among them four died and two others were treated with intensive chemotherapy followed by allogeneic stem cell transplantation. In the subgroup of patients exhibiting low percentage of BCL2L10 positive cells, one patient died of sepsis, and all others patients remained AZA sensitive according to the IWG 2006 or AML2003 criteria. Among these patients, 7 had a stable disease with hematologic improvement, 5 had a partial response and 11 patients had a complete response. The relative risk of this test using a cutoff of 50% BCL2L10 positive cells was estimated 11.5.

## DISCUSSION

BCL2L10 is a member of the Bcl-2 family that exhibits potent anti-apoptotic activity *in vitro*. Accordingly, BCL2L10 shares with the Bcl-2 protein family the well-conserved BH4, BH1 and BH2 domains and lack the BH3 domain that is characteristic of pro-apoptotic members of the Bcl2 family[25, 26]. BCL2L10 can interact with other members of the Bcl-2 family including Bcl-2, Bcl-xL and Bax to regulate apoptosis in different contexts[25, 27, 28]. Accordingly to its anti-apoptotic function, overexpression of BCL2L10 has been reported to suppress apoptosis through inhibition of cytochrome C release from mitochondria[26].

In a recent report, Fabiani et al reported that Decitabine another hypomethylating agent triggers apoptosis and up-regulation of several genes including BCL2L10[29]. Moreover, in primary samples from patients they found that BCL2L10 promoter was methylated in approximatively one-half AML and related myeloid neoplasms, 13% of MDS samples and in none of the controls. In a more recent report the same group showed that a higher methylation rate of BCL2L10 was significantly associated to worse OS in patients enrolled into the GIMEMA MDS0205 multicenter trial. This was confirmed in a group of 27 HR-MDS patients, treated with AZA. Patients with high BCL2L10 promoter methylation status had also a lower probability of achieving complete responses. However, in both study the authors did not study the relation between methylation of the BCL2L10 promoter and modulation of expression of its mRNA or protein level[30]. Of note, in gastric cancer cells methylation of the BCL2L10 promoter seems to correlate with decreased expression of BCL2L10 protein level [31, 32]. Taking into account the discrepancies reported regarding the impact of BCL2L10 promoter methylation status, it appears of great interest to consider its protein rather than its mRNA expression, in agreement with a recent report by Beverly et al. [33].

When we looked for BCL2L10 protein expression either by Western Blot or using our cytometry-based assay, expression of BCL2L10 was correlated with resistance to AZA in vitro. Importantly, knockdown of BCL2L10 but not Bcl2 by specific siRNA sensitized SKM1-R to AZA-mediated apoptosis clearly demonstrating an anti-apoptotic role for BCL2L10 in our cellular model. This was expected taking into account the structural homology of BCL2L10 with anti-apoptotic members of the Bcl2 family and the known anti-apoptotic function of this protein. Our results are in very good agreement with two studies in the literature that linked resistance of cancer cell lines established from solid tumor and myeloid leukemia to various chemotherapeutic agents and overexpression of BCL2L10 both at the mRNA and protein level [34, 35]. In addition, it has been recently reported that BCL2L10 expression was increased in patients with Multiple Myeloma (MM)[36]. In MM, BCL2L10 overexpression is clearly associated with apoptosis inhibition since BCL2L10 knockdown by specific siRNA or targeting BCL2L10 function by a small peptidomimetic consistently increased cell death[19], in good agreement with our own findings.

Importantly, we also establish in the present study that the number of BCL2L10 positive cells in the bone marrow is predictive of AZA resistance. Moreover, the percentage of BCL2L10 expressing BM cells from either healthy subjects or low-risk MDS patients was very low, conversely to the one of AZA-R patients. We confirmed by Western Blot that increased expression of BCL2L10 was a hallmark of AZA-resistant patients even though BCL2L10 expression and BCL2L10 positive cell counts were variable from one resistant patient to another. Collectively, our findings are consistent with an anti-apoptotic role of BCL2L10 in MDS that impacts on the sensitivity to AZA. In this line, over-expression of BCL2L10 positive cells not only correlates with resistance to AZA *in vitro* but is also a predictive factor for resistance to AZA in MDS patients.

Finally, we also established using Kaplan-Meier representation that OS of patients with less than 50% of BCL2L10 positive cells in the bone marrow was significantly higher than that of patients with more than 50% BCL2L10 positive cells. This test was highly sensitive and highly predictive for the outcome of patients. Finally, we found that all patients who were initially sensitive to AZA had lower level of BCL2L10 positive cells in their BM but consistently increased this number when they became resistant to this treatment.

In conclusion, we propose a very convenient and simple flow cytometry-based assay to systematically quantify BCL2L10 positive cells in the bone marrow of MDS or AML patients either at diagnosis or during the normal course of the disease. Patients with more than 50% BCL2L10 positive cells in the bone marrow at diagnosis could be considered as not eligible for AZA treatment. In these patients alternative therapy targeting BCL2L10 should be proposed. In this regard, peptidomimetics including Gossypol, ApoG and antimycin A which are potent inhibitor of BCL2L10 should be tested.

## MATERIALS AND METHODS

### Reagents and antibodies

RPMI, DMEM and fetal calf serum (FCS) were purchased from Invitrogen (Villebon sur Yvette, France). Sodium fluoride, sodium orthovanadate, phenylmethylsulfonyl fluoride (PMSF), aprotinin, leupeptin, 5-Azacitidine were purchased from Sigma (Saint-Louis, MO, USA). Anti-BCL2L10, anti-Bcl-xL, anti-ERK and anti-rabbit antibodies were from Cell Signaling Technology (Beverly, MA, USA). Anti-Bcl-2, Peroxidase-conjugated anti-goat and Peroxidase-conjugated anti-mouse antibodies were from Dakopatts (Glostrup, Denmark). HSP-60 and Mcl-1 antibodies were from Santa Cruz Biotechnology (Heidelberg, Germany). Alexa Fluor 488 Donkey anti-rabbit antibody was from Invitrogen (Villebon sur Yvette, France).

### Cell lines

The human cell lines SKM1-S and SKM1-R have been described elsewhere[17] and were cultured in RPMI 1640 supplemented with 10% FCS, 50 U/ml penicillin, 50 mg/ml streptomycin, and 1mM pyruvate under 5% CO2 in a humidified incubator. The Kidney cell line HEK293 was cultured in DMEM supplemented with 10% FCS, 50 U/ml penicillin, 50 mg/ml streptomycin, and 1mM pyruvate under 5% CO2 in a humidified incubator.

### Quantification of BCL2L10 by flow cytometry

White blood cells from bone marrow samples were isolated by density centrifugation (Ficoll-Paque Plus). Cells were fixed by paraformaldehyde 3%, permeabilized with Triton 0,1% and incubated with an anti-BCL2L10 antibody. Cells were incubated with secondary donkey anti-Rabbit FITC-antibody. BCL2L10 protein expression was quantified by flow cytometry (FL2 channel).

### RNA isolation and Reverse Transcription Polymerase Chain Reaction (RT-PCR)

Isolation of RNA and RT–PCR has been described in detail elsewhere[18]. To confirm differential mRNA expression, BCL2L10 mRNA was quantified by RT–PCR using the following primers: Forward primer (5’-3’) was CCT-TCA-TTT-ATC-TCT-GGA-CAC-G and Reverse primer (5’-3’) was TTT-CAC-TCA-AGG-AAG-AGC-C. PCR products (30μl) were loaded onto 3% agarose gels and visualized under ultraviolet light after staining with ethidium bromide.

### Plasmid constructions and directed mutagenesis

Epitop tagged-pEGFP expression and tagged-Myc pcDNA3-BCL2L10 plasmids have been previously described[19]. HEK293 cells were transfected with plasmid using JetPEI (Polyplus Transfection, Brighton, UK) according manufacturer’s procedure.

### Western blot analysis

After stimulation with various effectors for 24 h, cells were harvested and lysed in buffer containing 1% Triton X-100 and supplemented with protease and phosphatase inhibitors (Roche Diagnostics). Lysates were pelleted, and 50 μg of protein were analyzed by SDS-PAGE. Blot quantification was performed with Image J Software.

### RNA interference

The si-RNAs used for BCL2L10 and Bcl-2 knockdown have been described previously[19]. Briefly, cells were transfected with a si-RNA directed against either BCL2L10, Bcl-2, or Luciferase (Invitrogen, Villebon sur Yvette, France) (50 nM) using an Amaxa nucleofector for SKM1 cell lines or using Lipofectamine RNAiMAX (Invitrogen, Villebon sur Yvette, France) for HEK293 cell line. Seventy-two hours after transfection AZA-S and AZA-R cells were treated with 1 μM AZA for 24h. BCL2L10 and Bcl-2 expression were analyzed by protein gel blot.

### Assessment of cell metabolism

Cell metabolism was determined with XTT assay[20]. Cells (20 x 10^3^ cells) were incubated in a 96-well plate for 24h with AZA 1μM at a final volume of 100 μl. After 24h, 50 μl of XTT reagent was added to each well. The absorbance of the formazan product, reflecting cell metabolism, was measured at 490 nm. Each assay was performed in quadruplicate.

### Propidium iodide (PI) staining

After stimulation with 1μM AZA for 24h, cells were washed with PBS and stained with propidium iodide. Fluorescence was measured using the FL3 channel of a fluorescent-activated cell sorter (Miltenyi cytometer)[21].

### Active caspase 3 staining

After stimulation with 1μM AZA for 24h, cells were washed and stained with an anti-active caspase 3 antibody. Fluorescence was measured using the FL2 channel of a fluorescent-activated cell sorter (Miltenyi cytometer)[22].

### Patients Samples

Two cohorts of patients were used for this study. In Cohort 1, fresh bone marrow samples were collected from 32 patients treated with AZA. All patients had diagnosis of MDS or AML with less than 30% of blasts and IPSS scoring intermediate-2 or high. The diagnosis of MDS or AML was based on standard WHO criteria[23]. Patients (Pts) were to receive AZA at the FDA/EMEA approved schedule (75mg/m^2^/d, 7d/ 4 weeks). Pts having received ≥ 1 cycle of AZA and who had bone marrow evaluation after ≥ 4 cycles, or who died or progressed before completion of 4 cycles were considered evaluable (the last 2 groups were considered as treatment failures). Responses were scored according to IWG 2006 criteria for MDS and to Cheson et al.[24] for AML. Median number of AZA cycles at evaluation was 10 (3-22). All patients under treatment with AZA were included in protocol NCT01210274 (www.clinicaltrials.gov). Informed consent was obtained for all patients. Cohort 2: 45 frozen samples from low-risk and high-risk MDS or AML patients were collected as part of an institutionally approved cellular sample collection protocol. Treatment of patients was the same as the one described for cohort 1.

### Statistical analysis

BCL2L10 protein expression in sensitive and resistant patients of both cohorts was studied by Student’s t-test. OS of patients from cohort 1 was measured from the date of BCL2L10 protein evaluation to either death from any cause or last follow-up. When patients were treated with consolidative allogeneic stem cell transplantation, the data were censored at the date of graft. Survival functions were estimated by the Kaplan-Meier method and compared by the log-rank test. Differences between the results of comparative tests were considered significant if the two-sided P value was less than 0.05. All statistical analyses were performed using SAS 9.13 (SAS Institute, Cary, NC).

## Supplementary Figures and Tables






