# Supplementary material for: BCL2L10 is a predictive factor for resistance to Azacitidine in MDS and AML patients
Source: Oncotarget. 2012 May 9;3(4):490–501. doi: 10.18632/oncotarget.481 (PMC3380582; doi:10.18632/oncotarget.481)
Supplement: Supplementary file 1 [file oncotarget-03-490-s001.pdf]

BCL2L10 is a predictive factor for resistance to Azacitidine in MDS and AML patients – Cluzeau et al

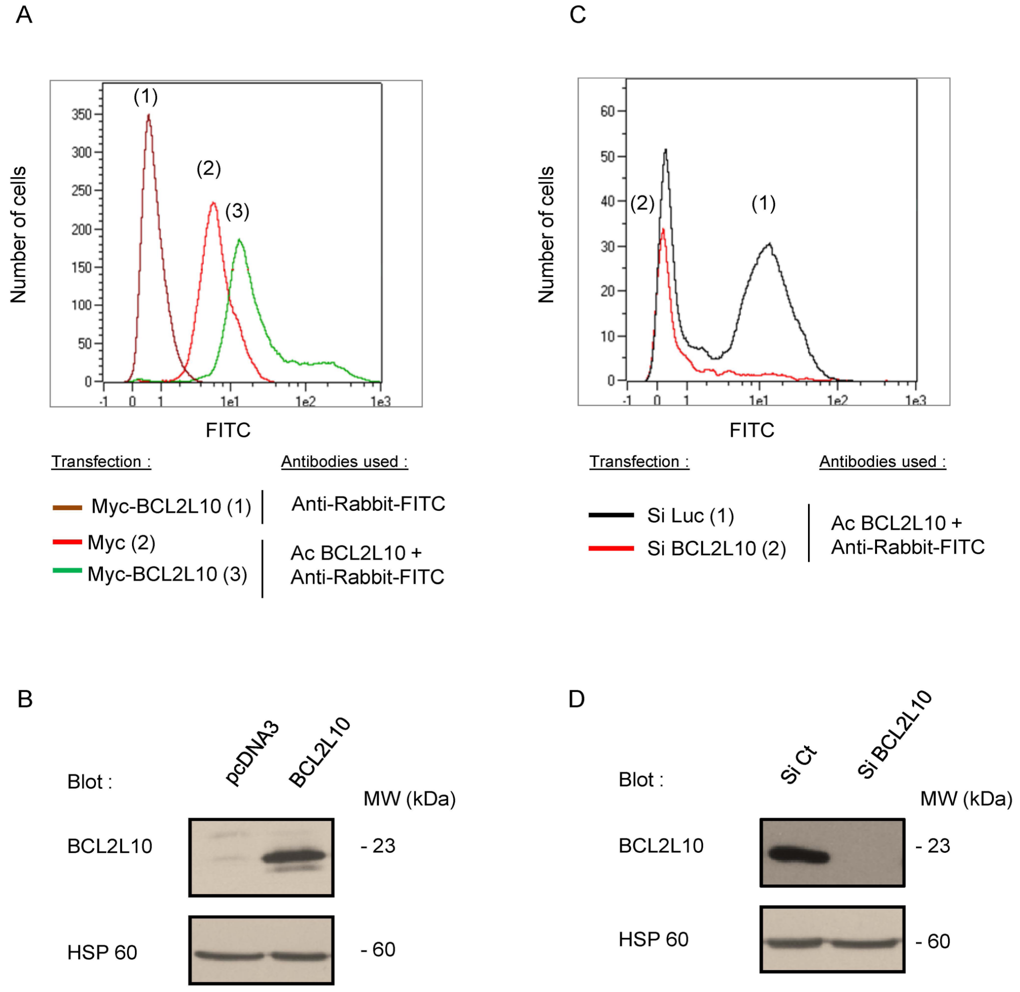

Supplemental Figure 1: Validation of flow cytometry quantification of BCL2L10 protein level. HEK293 cell line was used. (A) HEK293 cells were transfected with either pcDNA3 expression plasmids incorporating the N-terminal Myc epitope tag BCL2L10 or either pcDNA3 expression plasmids incorporating the N-terminal Myc epitope alone. Quantification of BCL2L10 protein level was performed by flow cytometry. (B) HEK393 cells were transfected with either si-Luc or either si-BCL2L10. Quantification of BCL2L10 protein level was performed by flow cytometry. (C,D) BCL2L10 protein level was vizualized by western blot. HSP was used as loading.

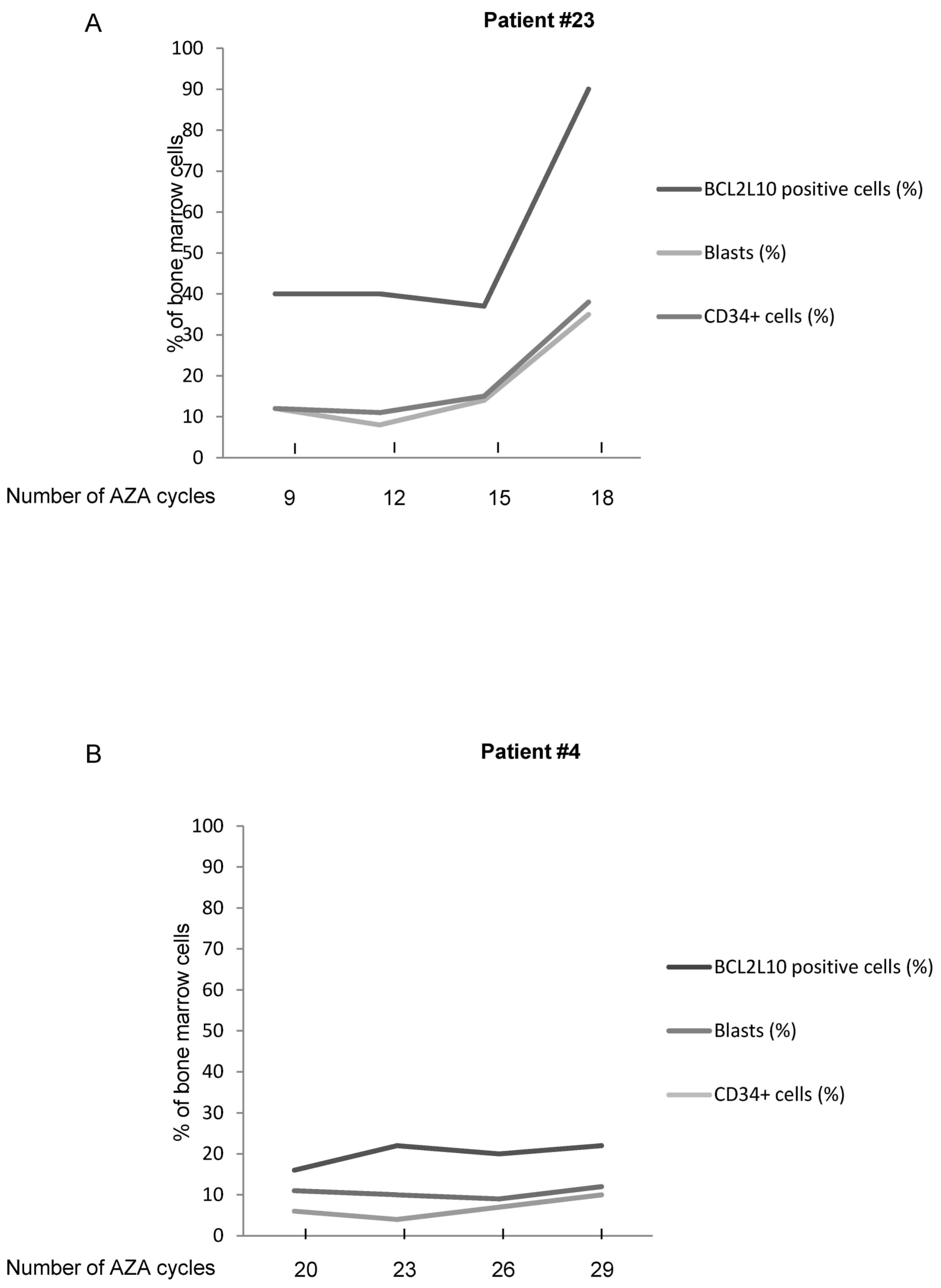

Supplemental Figure 2: Correlation between CD34+, blast, and high count of BCL2L10 positive cells and resistance to AZA in two MDS patients. The number of CD34+ cells, blasts and BCL2L10 expressing cells was determined in bone marrow samples from two patients after different cycles of AZA treatment. Patient #23 who became resistant increased his blast, CD34+ and BCL2L10 expressing cell number after 15 cycles while patient #4 maintained a low level of CD34+, blast and BCL2L10 positive cell counts.
